# Case Report: Management of Primary Tracheobronchial Light Chain Amyloidosis in a Patient With Biclonal Gammopathy Using a Systemic Bortezomib-Based Regimen

**DOI:** 10.3389/fmed.2021.728561

**Published:** 2021-10-15

**Authors:** Wei Yan, Peng Li, Cen Wu, Chuming Zhou, Aijun Liao, Wei Yang, Huihan Wang

**Affiliations:** ^1^Department of Hematology, Shengjing Hospital of China Medical University, Shenyang, China; ^2^Department of Respiratory Medicine, Shengjing Hospital of China Medical University, Shenyang, China

**Keywords:** light chain (AL) amyloidosis, tracheobronchial amyloidosis, biclonal gammopathy, dyspnea, case report, systemic chemotherapy

## Abstract

Primary tracheobronchial light chain (AL) amyloidosis is a rare and heterogeneous disease characterized by the buildup of amyloid deposits in the airway mucosa. Although its treatment remains challenging, the current view is that the localized form can be treated conservatively due to its slow progression. While radiotherapy has proven effective in treating localized form of the disease, some patients do not respond to local treatment and continue to experience poor quality of life, highlighting the need to explore additional treatment strategies. In this report, we discuss a case of primary tracheobronchial AL amyloidosis with biclonal gammopathy (IgA κ and IgG κ) in a 46-year-old man who was transferred to our hospital due to dyspnea progression over the preceding 3 years. Chest computed tomography revealed irregular tracheobronchial stenosis with wall thickening, and histological examination of the bronchial biopsies confirmed the diagnosis of endobronchial AL amyloidosis. Owing to the poor effect of radiation therapy and treatments for improving airway patency, he was treated with a systemic chemotherapy regimen [cyclophosphamide-bortezomib-dexamethasone (CyBorD)]. We observed substantial improvements in his dyspnea, highlighting the potential of systemic therapy to improve quality of life of patients with tracheobronchial AL amyloidosis. However, the long-term pathological changes associated with local bronchial lesions require further investigation.

## Introduction

Light chain (AL) amyloidosis is an uncommon, heterogeneous disease characterized by the deposition of immunoglobulin light chain amyloid fibrils, leading to functional impairment of the affected organs (1). In its localized form, AL amyloidosis is limited to single organs such as the gastrointestinal tract, bladder, skin, or head and neck (2). However, AL amyloidosis with localized deposition in the lower respiratory tract is rather unusual, and the associated symptoms and epidemiologic characteristics are highly non-specific, occasionally mimicking different pathologies such as asthma or chronic bronchitis (3). Thus, primary tracheobronchial AL amyloidosis can present a substantial diagnostic and therapeutic challenge.

Previous studies have reported that the incidence of biclonal paraproteinemia is ~1.5–4% of that reported for monoclonal gammopathies, particularly for monoclonal gammopathies of undetermined significance (MGUS). Biclonal gammopathies are relatively common among patients with multiple myeloma (MM), non-myelomatous malignant diseases, and cancer (4–6); primary AL amyloidosis with biclonal paraproteinemia is even rarer. Given that tracheal amyloidosis is also relatively rare, there are currently no clear guidelines for treatment. Nonetheless, local resection and radiotherapy are the mainstays of treatment due to the slowly progressive nature of local amyloidosis. However, there is an urgent need to improve treatment efficacy and the quality of life of patients in whom local treatments have failed as well as those with severe tracheal symptoms. In the present report, we discuss a case of primary tracheobronchial AL amyloidosis characterized by biclonal paraproteinemia. Although the patient did not respond to local treatment for tracheal amyloidosis, we achieved a good curative effect *via* systemic treatment, highlighting the potential of this treatment strategy for patients with similar diseases.

## Case Report

The patient was a 46-year-old man with a history of recurrent cough, expectoration, and a 3-year history of progressive dyspnea. Before admission to our hospital, he was diagnosed with chronic bronchitis and bronchiectasis at other hospitals and had received antibiotic treatment numerous times. Despite antibiotic treatment, his symptoms progressively worsened such that he required oxygen therapy and ECOG performance status of him was 4. A physical examination revealed that his respiratory rate was 25 cycles per minute. Moreover, diffuse wheezing could be heard in both the lung fields. Laboratory findings included leukocytosis (11.52 × 10^9^/L) and significantly reduced partial pressure of oxygen (63 mmHg). Arterial blood gas analysis revealed an oxygen uptake of 3 L/min. We also have conducted related inspections to rule out systemic involvement. Results of tests for liver and kidney function, myocardial enzyme levels, NT-proBNP, and troponin levels were unremarkable, and no proteinuria was found on 24-h urine analysis. Color Doppler echocardiography showed normal cardiac structure. No abnormalities were found in gastrointestinal endoscopy. The results of the PPD test were negative, as were all sputum samples for bacteria, acid-fast bacilli, and fungi. The patient's lung function test revealed obstructive dysfunction of pulmonary ventilation with a severe reduction in small airway function, and his bronchodilator test result was negative.

**Figure 1:**
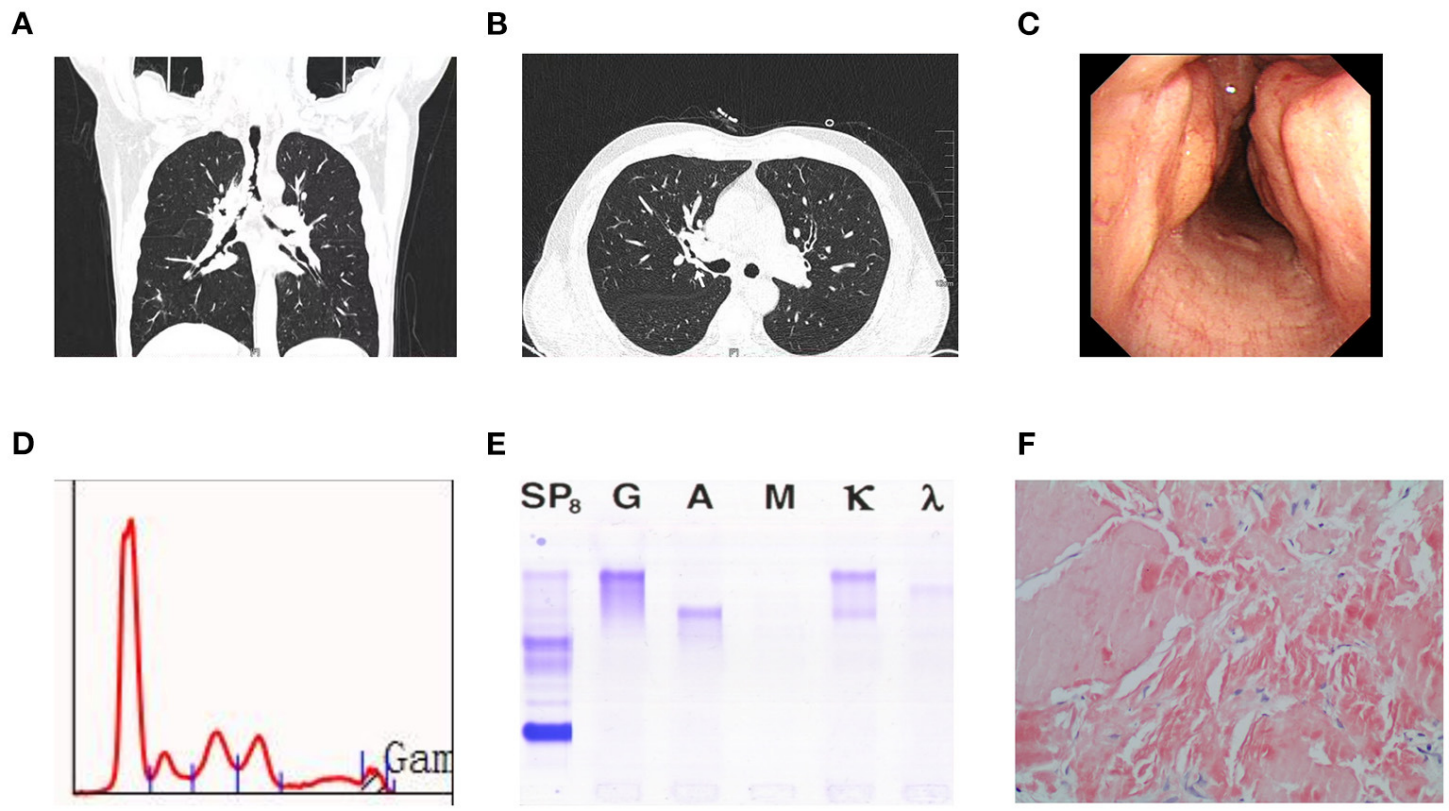
**(A)** Computed tomography scan revealed uneven thickening of the walls of the trachea and bronchi at different levels with luminal narrowing. **(B)** Computed tomography scan showed obliteration in the right intermediate bronchus, but pulmonary parenchyma were normal. **(C)** Bronchoscopy revealed roughness of mucosa in trachea and multiple nodular in airway lumen. **(D)** Serum protein electrophoresis showing an M-peak in the gamma fraction. **(E)** Serum immunofixation electrophoresis. It shows biclonal gammopathy of IgG kappa type and IgA kappa type. **(F)** Amyloidosis was confifirmed by tissue biopsy, which showed Congo red dye staining of lesion was positive (Hematoxylin and Eosin, × 200).

Chest computed tomography (CT) revealed that the tracheal walls and bronchi were extensively uneven to different degrees and exhibited luminal stenosis (Figures 1A,B). Electronic bronchoscopy revealed completely irregular and thickened bronchial mucosal surface with prominent nodes accompanied by a small amount of white secretion (Figure 1C). The lumen of the right middle bronchus exhibited extreme narrowing, and pathological examination of the tracheal mucosal biopsy specimen at the site of narrowing revealed mucosal calcification and fibrous hyperplasia with deposition of kappa light chain, as well as the following: PAS stain (–), acid-fast staining (–), and Congo red stain (+) (Figure 1F).

Serum electrophoresis showed a monoclonal spike, although immunofixation electrophoresis (IFE) revealed a double monoclonal component: IgA κ and IgG κ (Figures 1D,E). Serum immunoglobulin A (IgA), IgM, and IgG levels were 1.48 g/L (0.7–4.0 g/L), 0.62 g/L (0.4–2.3 g/L), and 5.23 g/L (7.0–16.0 g/L), respectively. The free κ and λ light chain levels were 15.8 mg/L (normal, 6.7–22.4 mg/L) and 17.8 mg/L (normal, 8.3–27.0 mg/L), respectively, with a normal κ/λ ratio of 0.888 (normal, 0.31–1.56). Bone marrow cytology revealed an irregular monoclonal plasma cell population (CD45+, CD38+, CD138+, CD200+, and ckappa+), representing 0.86% of the total leukocytes.

The patient underwent further examinations to determine the extent of systemic involvement. Echocardiography revealed no signs of myocardial involvement. Skeletal osteolytic lesions, organomegaly, and neuropathy were absent. Blood biochemistry revealed normal levels of creatinine, liver enzymes, and alkaline phosphatase. Based on the comprehensive evaluation described thus far, the patient was diagnosed with localized amyloidosis given the observed lack of extrapulmonary organ involvement.

The broad endobronchial manifestations enabled the performance of endoscopic resection of the amyloid masses under combined rigid and soft bronchoscopy guidance. The patient underwent rigid bronchoscopy with argon plasma coagulation (APC) and Nd:YAG laser debridement for the treatment of his endobronchial obstruction. However, this did not improve the patient's dyspnea with massive expectoration. Therefore, we attempted to relieve his persistent endobronchial obstruction by initiating external beam radiation therapy (EBRT) at a dose of 20 Gy in 10 fractions of 2 Gy each. However, the patient's dyspnea showed no significant improvement at 3 months after the EBRT. Another bronchoscopy revealed progression of the tracheobronchial lesions due to the endobronchial situation. Subsequently, he experienced an aggravation of dyspnea, developed an inability to move, and reported poor quality of life.

The patient then consulted with our hematology department. Although he had not previously received systemic treatment for local amyloidosis, we noticed that local treatment could not alleviate his symptoms or delay the disease progression. Therefore, given his strong desire for treatment, we administered systematic chemotherapy. He was treated with one cycle of CyBorD [subcutaneous (SC) bortezomib: 1.3 mg/m^2^ on days 1, 4, 8, and 11; intravenous (IV) cyclophosphamide: 300 mg/m^2^ on days 1–4; IV dexamethasone: 20 mg on days 1–4 and 8–11]. Interestingly, the patient's symptoms of dyspnea improved significantly after treatment. In addition to a decrease in sputum volume, he began to perform simple daily activities, and evidence suggested functional improvements in oxygenation and spirometry findings (Table 1). However, follow-up bronchoscopy and chest CT performed 1 month later revealed no improvements in the obstruction. After one cycle of treatment, there was minimal secretion, the patient did not require inhaled oxygen, and he could walk independently. The patient has completed three cycles of treatment, and the ECOG performance status is 0. The patient exhibits good tolerance to CyBorD regimen, and no treatment-related adverse reactions such as peripheral neuritis, infectious disease and bone marrow suppression have occurred. The patient's ECOG score is reduced from 4 to 0 which shows an improvement of life quality, and the pulmonary function test results show that obstructive ventilatory disorder is improved. The patient is evaluated a validated organ response.

**Table 1:** Pulmonary ventilation function test before and after CyBorD chemotherapy.

	**Before CyBorD chemotherapy**	**After CyBorD chemotherapy**
VC (L)	2.47 (52.1% pred.)	4.37 (95.3% pred.)
FEV1 (L)	0.70 (18.9% pred.)	2.29 (63.8% pred.)
FEV1/FVC (%)	0.28	0.52

*CyBorD, cyclophosphamide-bortezomib-dexamethasone; VC, vital capacity; FEV1, forced expiratory volume in 1 s*.

## Discussion

AL amyloidosis can be classified as systemic or local based on the distribution of abnormal amyloid deposits (7). Systemic amyloidosis is initiated by light chains produced by B-cell clones in the bone marrow, which may target multiple organs. In contrast, localized AL amyloidosis is an extremely rare and less studied disease in which locally produced light chain deposits form at a single anatomic site, appearing as one or multiple tumor-like amyloid lesions (8). A large body of data shows that localized AL amyloidosis may contribute to dysfunction in multiple organs that are not often affected by systemic amyloidosis, such as the breasts, gastrointestinal tract, urinary tract, and others (9–12). However, involvement of the respiratory system is rare. Tracheobronchial amyloidosis is the most common subtype of primary lower respiratory tract AL amyloidosis (13). Symptoms are non-specific (e.g., dyspnea, wheezing, coughing, and hemoptysis), which can lead to delayed diagnosis or misdiagnosis of another respiratory illnesses.

Typically, there are no circulating monoclonal immunoglobulins in the serum or urine of patients with localized tracheobronchial AL amyloidosis, and there is no evidence of bone marrow involvement for clonal plasma cells (14). Our patient's IFE findings were positive, and he presented with biclonal gammopathy, with normal serum levels of free light chains and immunoglobulins. Moreover, malignant monoclonal plasma cells were also observed in the bone marrow. While MGUS may progress to systemic amyloidosis or myeloma, this does not seem to occur in patients with localized amyloidosis (15). Thus, our patient was diagnosed with primary localized tracheobronchial AL amyloidosis.

Patients with double monoclonal gammopathic manifestations (DMGMs) can express two different M proteins that differ based on the presence of heavy chain (HC) isotypes with the same light chains (LCs) or LC isotypes with the same HC isotype (16). Our patient exhibited DMGMs (IgA κ and IgG κ). The clinical manifestations and therapeutic responses of patients with DMGMs has been reported to be similar to those observed in patients with monoclonal gammopathic manifestations (MGMs) (17). Nevertheless, when compared with MM and lymphoproliferative diseases, DMGMs associated with AL amyloidosis are extremely rare. Kyle et al. reported the first five cases of AL idiopathic amyloidosis in patients with biclonal gammopathy in 1981 (18), following which several similar cases were reported (19, 20). According to the Amyloidosis Research Center at the University of Pavia, a comparable proportion (6%) of cases with a biclonal gammopathy, detectable *via* serum and urine IFE, was observed among 868 patients with AL amyloidosis evaluated from 1986 to 2007 (21). Tschumper et al. reviewed the clinical data of patient samples in the Mayo Clinic monoclonal gammopathies tissue bank, identifying three patients with AL amyloidosis associated with DMGs (22). Among the 539 patients with biclonal gammopathy investigated in a 2016 Mayo Clinic study, 25 had AL amyloidosis (23). The present case and existing research suggest that DMGMs in patients can aid in monitoring therapeutic responses and guide treatment modification during the follow-up period.

There is no established treatment for localized tracheobronchial AL amyloidosis. Due to the scant number of cases, no randomized trials have been performed. In their analysis of 293 patients with localized AL amyloidosis (locAL), Basset et al. reported lower respiratory tract involvement in 10.6% of the cases, as well as a 5-year progression free survival rate of 80%. In their study, 16 patients died during a median follow-up of 44 months, all of whom exhibited respiratory tract involvement (nine with lung involvement, four with lower airway involvement, and three with locAL in the nasopharynx) (24). Local tracheobronchial AL amyloidosis seems to be heterogeneous, with symptoms and concomitant disorders having the potential to affect prognosis. Therefore, cases should be managed on an individual basis. Due to its slowly progressive nature, a “watch and wait” strategy can be adopted in asymptomatic cases. Surgical resection, balloon dilation, stenting, APC, laser treatment, electrocautery, and tracheostomy have also been proposed as alternatives for the treatment of symptomatic patients (25). Although prognosis is more favorable for locAL than for systemic amyloidosis, some patients with the former experience relapse. A recent report described a case that was successfully treated with EBRT (26). Several other reports have demonstrated that, after EBRT treatment, patients receiving other therapies exhibit symptomatic, bronchoscopic, and functional improvement (27, 28). Such studies suggest that EBRT is not only safe but may also provide both symptomatic and objective improvement (29).

Considering the severity of the respiratory obstruction in our patient, we first adopted APC and Nd:YAG laser debridement to alleviate his respiratory symptoms. However, his clinical symptoms and imaging findings did not improve, and the presence of inflammation further aggravated his dyspnea and expectoration. Although we subsequently initiated EBRT, his symptoms of dyspnea failed to improve, and follow-up bronchoscopy indicated persistence of diffuse airway stenosis. Although pharmacologic treatments, including corticosteroids, melphalan, and colchicine, have been in use for many years, they have been associated with no apparent benefits. Because we identified malignant plasma cells in the bone marrow of our patient and since his IFE findings were positive, we speculated whether treatment for systemic amyloidosis would be effective. The CyBorD regimen (cyclophosphamide-bortezomib-dexamethasone) has become the standard of care for treating systemic amyloidosis, recommended by guidelines in various countries (30), with a reported median time to organ response of 2–3 months (31). However, the apparent relief of the patient's symptoms and his improvement in lung function could not be explained by anti-infective therapy alone. Indeed, the need for additional chemotherapy and the timing of the next chemotherapy treatment remain controversial.

In conclusion, primary tracheobronchial AL amyloidosis with biclonal paraproteinemia is a rare disease entity whose pathogenesis requires further exploration. Treatment options for this disease have ranged from observation alone to aggressive local and systemic therapy. Individualized treatment is recommended, and further research is required to develop more effective treatment strategies.

## Data Availability Statement

The raw data supporting the conclusions of this article will be made available by the authors, without undue reservation.

## Ethics Statement

The studies involving human participants were reviewed and approved by the Ethics Committee of China Medical University's Shengjing Hospital. The patients/participants provided their written informed consent to participate in this study. Written informed consent was obtained from the individual(s) for the publication of any potentially identifiable images or data included in this article.

## Author Contributions

HW and WYan designed the study, performed the histological evaluation, and drafted the manuscript and figures. WYang and AL analyzed the amyloid deposits and reviewed the manuscript. PL, CW, and CZ participated in the treatment, evaluated the clinical data, and reviewed the manuscript. All authors contributed to the article and approved the submitted version.

## Conflict of Interest

The authors declare that the research was conducted in the absence of any commercial or financial relationships that could be construed as a potential conflict of interest.

## Publisher's Note

All claims expressed in this article are solely those of the authors and do not necessarily represent those of their affiliated organizations, or those of the publisher, the editors and the reviewers. Any product that may be evaluated in this article, or claim that may be made by its manufacturer, is not guaranteed or endorsed by the publisher.
