# Supplementary material for: Case Report: Management of Primary Tracheobronchial Light Chain Amyloidosis in a Patient With Biclonal Gammopathy Using a Systemic Bortezomib-Based Regimen
Source: Front Med (Lausanne). 2021 Oct 15;8:728561. doi: 10.3389/fmed.2021.728561 (PMC8554224; doi:10.3389/fmed.2021.728561)
Supplement: Supplementary file 1 [file Table_1.docx]

Supplementary Table 1. A timeline with relevant data from the episode of care.

| Time | Event |
| --- | --- |
| 2020.09.12 | Chest computed tomography  Electronic bronchoscopy  Lung function test |
| 2020.09.28 | Tracheal mucosal biopsy  diagnosis |
| 2020.10.08 | [Local](D:/Program%20Files%20(x86)/Youdao/Dict/8.9.6.0/resultui/html/index.html#/javascript:;) [radiotherapy](D:/Program%20Files%20(x86)/Youdao/Dict/8.9.6.0/resultui/html/index.html#/javascript:;) |
| 2021.04.14 | Bronchoscopic physical therapy |
| 2021.05.04 | Bone marrow tests  Serum electrophoresis  Free κ and λ light chain tests |
| 2021.05.12 | CyBorD treatment |
| 2021.06.22 | Bronchoscopy  Chest CT  Lung function test |
| 2021.06.30 | CyBorD treatment |
| 2021.08.15 | CyBorD treatment |
